# COVID‐19 and community‐based care services: Experiences of people living with dementia and their informal carers in Italy

**DOI:** 10.1111/hsc.13758

**Published:** 2022-02-20

**Authors:** Ilaria Chirico, Giovanni Ottoboni, Clarissa Giebel, Alessandro Pappadà, Marco Valente, Valentina Degli Esposti, Mark Gabbay, Rabih Chattat

**Affiliations:** ^1^ 9296 Department of Psychology University of Bologna Bologna Italy; ^2^ 4591 Department of Primary Care & Mental Health University of Liverpool Liverpool UK; ^3^ NIHR ARC NWC Liverpool UK

**Keywords:** caregivers, COVID‐19, dementia, public health, social care, social support, technology

## Abstract

The COVID‐19 pandemic has significantly limited access to health and social care support systems for people with dementia and their carers, compounding the severe social restrictions. The aim of this study was to investigate the experiences of COVID‐19 among community‐dwelling people with dementia and their informal carers in Italy. Specifically, we focused on access to community‐based services and adopted solutions to provide support and care during exceptional times. Informal carers, caring for someone with dementia and attending community‐based services in Italy, participated in remote semi‐structured interviews between October and November 2020. Participants were asked about the effects of social isolation and closure of in‐person services on their daily lives as well as the challenges of dementia care. Transcripts were analysed using inductive thematic analysis. 22 informal carers were interviewed. Three themes emerged: (1) Disruptions to people with dementia's lives and health; (2) COVID‐19 as an additional stressor for carers; and (3) New ways of caring for people with dementia during COVID‐19. Face‐to‐face social care and social support services were suddenly interrupted and restrictions on social distancing were introduced, thus leading to people with dementia's impaired health and increased behavioural and psychological symptoms. Not only the amount but also the intensity of care increased, with no chance of respite for informal carers. Overall remote activities provided participants with emotional and social benefits, while allowing the continuity of relationships with services staff and users and of care. However, according to carers, a combination of virtual and face‐to‐face activities could better counterbalance the multiple adverse outcomes of COVID‐19. Public health measures should be designed carefully to consider the safety needs and the physical, psychological and social needs of people with dementia. Within a holistic care approach, social care services need to be enabled better to guarantee high‐quality care even during pandemic times.

**What is known about this topic?**

- The COVID‐19 pandemic significantly reduced access to health and social care support systems for people with dementia and their informal carers.
- Social care support services offer a range of physical, cognitive and social activities that help people with dementia to live well with their condition while delaying their institutionalisation.
- The interruption of in‐person social care support services along with restrictions on social distancing led to the worsening of people with dementia's symptoms and increased carer burden.

**What this paper adds?**

- This is the first study to explore if and what alternative forms of support and care have been implemented by community‐based services following the pandemic outbreak in Italy.
- Different types of remote activities were introduced nearly 2 months after the pandemic outbreak. They provided participants with emotional and social benefits, while allowing the continuity of relationships with services staff and users and of care.
- Remote activities did not compensate for face‐to‐face activities and, therefore, social care support services need to be enabled better to guarantee high‐quality care even during pandemic times.

## 1 INTRODUCTION

There is mixed evidence that the COVID‐19 pandemic and related public health measures have negatively impacted on the mental health of the general population, thus increasing referrals for acute panic, anxiety, obsessive behaviour, hoarding, paranoia, depression, and post‐traumatic stress disorder (Brooks et al., 2020; Luo et al., 2020). A more recent systematic review suggests that levels are falling back towards pre‐pandemic norms, although study findings are heterogeneous (Robinson et al., 2022). However the evidence still points to the most vulnerable groups of society, such as people living with dementia, who are at increased risk of severe COVID‐19 infection, morbidity, and mortality having been most adversely affected (Manca et al., 2020).

Worldwide, there are more than 55 million people living with dementia, of whom an estimated 14.1 million live in Europe (World Health Organization, 2021), and more than 1 million in Italy (Alzheimer Europe, 2019). The latter was the first European country affected by the pandemic, and the first one in the world to impose a national lockdown on March 9, 2020. With social distancing rules implemented across the world, home visits from relatives and/or friends, as well as social activities, were significantly reduced or completely stopped (Mok et al., 2020). The health care sector was also affected by the pandemic, thus leading to a lack of provision of ambulatory services, day clinics and prescribed therapies, as well as social support (Giebel, Lord, et al., 2021; Giebel, Pulford, et al., 2021; Thyrian et al., 2020). It is well known that social support services (e.g., day care centres, peer support groups, respite care) provide a daily routine that helps to decrease the behavioural and psychological symptoms of people living with dementia, thus delaying their institutionalisation (Orellana et al., 2020). Furthermore, social support activities are associated with people living with dementia's decreased loneliness and depression, and higher quality of life (Orellana et al., 2020).

To date, there is increasing evidence that COVID‐19 containment measures led to the deterioration of physical and cognitive health conditions, and to the onset or worsening of behavioural and psychological symptoms (e.g., agitation/aggression, apathy, depression) of people living with dementia (Cagnin et al., 2020; Numbers & Brodaty, 2021; Talbot & Briggs, 2021). As a consequence, the adjustment of ongoing pharmacological therapy or the introduction of new treatments, mostly antipsychotics, was sometimes required (Canevelli et al., 2020).

In the context of COVID‐19 strict limitations and the temporary closure of in‐person services, informal carers felt anxious about potential negative consequences on their relative's health and experienced a lack of respite due to decreased support and extra‐care responsibilities (Giebel, Cannon, et al., 2021; Hanna et al., 2021). It was common for them to repeatedly explain and/or remind their relatives to follow the COVID‐19 protective guidelines, such as wearing face masks and/or to entertain them as best they could (Devita et al., 2020). Hence, the pandemic often represented an additional stressor for informal carers, thus exacerbating the difficulties they already had to face daily as primary care providers (Chirico, Ottoboni, Linarello, et al., 2021; Chirico, Ottoboni, Valente, et al., 2021).

Overall, these emerging results highlight how the experience of confinement may have triggered a vicious cycle with detrimental effects on the health and quality of life of both people living with dementia and their informal carers (Manca et al., 2020; Rainero et al., 2021). In this context, increasing attention has been paid to those alternative measures capable of mitigating the psychosocial implications of the pandemic. Among them, digital technology may compensate for the lack of face‐to‐face care provision, thus allowing the continuity of care and social connectedness, while decreasing exposure to individual risk and the pressure on health and care systems (Goodman‐Casanova et al., 2020; Pappadà et al., 2021).

This study, as part of a larger international study, explored the experiences of COVID‐19 of community‐dwelling people living with dementia and their informal carers in Italy. To our knowledge, no studies have explored if and what alternative forms of support and care have been implemented by community‐based services (i.e., meeting centres, Alzheimer's Cafés) after the pandemic outbreak in Italy. Findings of this study will inform researchers, practitioners and commissioners in the field of dementia care to develop guidance for recovery and remedial measures as services are reintroduced and ways to mitigate these negative impacts. Specifically, we wanted to investigate potential new ways of effectively caring and supporting people living with dementia to live well with their condition. These solutions may be implemented for potential future public health emergencies as well as for enhanced care integration, quality improvement and effective targeting.

## 2 METHODS

### 2.1 Participants and recruitment

The sample consisted of informal carers (i.e., family members), aged 18+, caring for their relative living with dementia and residing in the community. Informal carers were recruited via third sector and social support organisations located in Northern Italy. Upon receiving a list of potential participants, research team members called these to discuss the study and arrange the interview, if interested.

Recruitment stopped when the point of saturation was met, i.e. no additional themes emerged during the analysis of three consecutive interviews (Saunders et al., 2018). Ethical approval was obtained from the Ethic Committee of the University of Bologna (Italy) [Ref: 41453] prior to study commencement.

### 2.2 Data collection

Data were collected remotely by telephone interviews between October 2020 and November 2020. During this period, in order to contain the spread of COVID‐19, Italian regions were divided into risk‐level zones: red for the highest, followed by orange and yellow. Data collection occurred within the yellow zone (i.e., the lowest risk classification), where standard infection risk‐reduction rules applied^1^ and there were no further restrictions such as travel bans, commercial/foodservice activities suspended.

Informal carers provided written informed consent via email before the start of the interviews. Sociodemographic characteristics about participants and their relatives living with dementia (e.g., gender, age, type of relationship), as well as information about the diagnosis (dementia subtype and years since diagnosis) were collected.

The interview guide was developed with informal carers, a person living with dementia, professionals, and social support service providers in the UK (i.e., lead research group for this international study). Then, this was adapted culturally and translated into Italian.

Interviews lasted around 45 min (range: 30–60). Participants were encouraged to describe their experiences with dementia services before and since the start of COVID‐19, as well as the impact of social distancing rules on people living with dementia and on their own daily lives. Discussions also explored the alternative support provided by services during the pandemic. In line with the semi‐structured interview format, the interviewer asked participants to add some details or to clarify their answers, if necessary.

### 2.3 Data analysis

Audio recordings of the interviews were transcribed verbatim and analysed using inductive thematic analysis (Braun & Clarke, 2006). Data analyses were undertaken by two researchers (I.C., V.D.E.) independently. After having read the interviews repeatedly, they used a feed‐forward strategy to identify the main content units. These were summarised in codes and, then, grouped into subthemes and themes by each researcher. The consistency of the clustering process was discussed after each interview, and any disagreements were resolved through a consensus discussion with the remaining study authors (Hickey & Kipping, 1996).

## 3 RESULTS

### 3.1 Background characteristics

Twenty‐two interviews were undertaken with adult children of people living with dementia. Their ages ranged between 37 and 80 years (*M* = 58.3, *SD* = 9.4). The majority of carers were female (81.8%) and married (77.3%). They had predominantly completed their education at the end of secondary (77.3%), or middle (13.6%) school, while a smaller percentage obtained an academic degree (9.1%). At the time of the interview, more than half (68.2%) of carers were in paid employment and did not cohabit with their parent living with dementia (63.6%).

People with dementia were on average 83.5 (*SD* = 6.2) years old (range 70–93) and mostly female (90.9%). Dementia subtypes were Alzheimer's disease (54.6%), followed by vascular and mixed dementia (13.6%; 9.1%), and the remaining were unknown (22.7%). The mean number of years since diagnosis was 4.8 (*SD* = 2.9; range 1–10) years. Nearly half (45.4%) of people living with dementia attended a meeting centre, or attended Alzheimer's Cafés (36.4%), or both of them (18.2%).

### 3.2 Qualitative findings

Thematic analysis identified three overarching themes across the interviews (Table 1): (1) Disruptions to people with dementia's lives and health (two subthemes); (2) COVID‐19 as additional stressor for carers (two subthemes); and (3) New ways of caring for people with dementia during COVID‐19 (three subthemes). Each theme is described below, with verbatim extracts of participants' responses.

**TABLE 1:** Themes and subthemes from interviews

Themes	Subthemes
1. Disruptions to people with dementia's lives and health	Faster cognitive and physical decline
	Increased/worsened behavioural and psychological symptoms
2. COVID‐19 as additional stressor for carers	High carer emotional strain in response to COVID‐19 measures Sudden service closures and increased carer burden
3. New ways of caring for people with dementia during COVID‐19	Adaptation of community‐based services in a digital format Perceived effects of remote care and support Unmet care needs

#### 3.2.1 Theme 1: Disruptions to people with dementia's lives and health

##### Faster cognitive and physical decline

Participants reported that people living with dementia's symptoms worsened during COVID‐19. Specifically, they noticed a deterioration in cognitive abilities, especially temporal and spatial orientation, which were linked with the disruption of daily routine, lack of stimuli and staying all day at home.

It is very hard for her to understand the difference between morning and evening now. When it's night, she says "it's already night" or she even mistakes the afternoon for the morning. (ID5, daughter, 60 years)

Similarly, people living with dementia's motor skills such as balance and coordination worsened more quickly than expected. Participants believed that this was due to physical inactivity and sedentary behaviours linked to public health restrictions on freedom of movement and gatherings. Due to the closure of community‐based services and restrictive measures, physical exercise routines were disrupted and even low‐level physical activities, such as going out and walking, suddenly stopped.

She was certainly less stimulated and this implied a gradual physical loss. Maybe it was part of the natural decline, but the lack of an adequate stimulation made it worse. On a motor level, she certainly changed, previously she was able to walk alone, now we are using the medical walker because she has not the same equilibrium as before. (ID13, daughter, 60 years)

##### Increased/worsened behavioural and psychological symptoms

According to carers, the multiple disruptions to people with dementia's lives and the prolonged isolation led to increased behavioural and psychological symptoms. Carers’ strategies to manage them, such as going out for a walk, were no longer feasible due to environmental restrictions. The most commonly reported symptoms were: apathy/lethargy, low mood, lack of appetite, agitation/irritability. People living with dementia could find it difficult to engage in any activity, even dressing sometimes, increasingly being withdrawn and unresponsive to usual stimuli. They sometimes stopped initiating conversation, and could also appear agitated and react negatively to pandemic restrictions and rules, such as wearing a mask. This overall contributed to a tense domestic atmosphere compounding the pressure on carers.

The lack of sociality massacred him. As I mentioned you, he was a very friendly and sociable person, and since the lockdown he changed a lot, he lost a lot, his interests as well. At the beginning, I thought it was depression because he was watching the television all time, he stopped using the telephone and no longer called, previously he called a lot … in a few words, few stimuli. My mum always had to tell him «do this, call, move, take a walk». (ID18, daughter, 48 years)

#### 3.2.2 Theme 2: COVID‐19 AS AN additional stressor for carers

##### High carer emotional strain in response to COVID‐19 measures

Participants reported that the atmosphere at home felt restricted, like a prison, and was accompanied by increasing feelings of emotional distress, anxiety and uncertainty/loss of control. Many participants felt that time had suddenly stopped, and this was harder to manage for those who did not work.

We limited contacts a lot and it is very harmful for us, since we talk less and less to anyone, by telephone to some relatives and friends. I miss sociality, we only go out to go shopping and that's it. I’m particularly affected by this situation because I don't have a job. It is as if we had stopped, suspended ourselves waiting for better times. (ID4, daughter, 63 years)

Informal carers paid great attention toward restrictive measures, as they were seriously concerned about their relative's health. To reduce the risk of contagion, they avoided any contact with the outside world and went out only if necessary (i.e., to buy food or medicines). This exacerbated their feelings of loneliness and being emotionally overwhelmed, even more so when the domestic situation was also deteriorating (see above).

The fear of COVID‐19 was overwhelming more than anything else, by avoiding any contact for a possible transmission and taking all precautions rather than just being isolated, since we still didn't know how the contagion took place, so we were scared, frightened. (ID6, son, 52 years)

##### Sudden service closures and increased carer burden

Informal carers felt abandoned due to the sudden closure of health and social care services, the cancellation of specialist visits, and the fear of not being able to rely on professionals as before. As a consequence of the lack of formal care, they tried to engage their relatives as much as they could do. However, they felt quite unprepared and mentioned having many difficulties in doing it properly.

We felt a bit abandoned because we couldn't go to the meeting center. It took away the chance to stimulate my mum, so we honestly felt a bit unprepared, we found it very hard to stimulate her. (ID5, daughter, 60 years)

Moreover, the limited domestic environment was a matter of concern during the pandemic as it was challenging to reinvent the daily routine, trying to manage every day differently, to introduce variety and stimuli where they could. Participants often spent more hours with their relative and this increased carer burden, due to both the lack of time for themselves and a more challenging caring routine associated with the worsening of their relative's symptomatology. They felt exhausted as they had to repeat over and over again the information about COVID‐19 to their loved one with dementia. Even those few participants who employed a carer were concerned about the situation at home, due to the difficult management of their relative's behavioural and psychological symptoms as well as to the paid carer's worries about COVID‐19.

I’m psychologically overwhelmed. Spending all day with my mum to whom I have to repeat the same things over and over again. I’m very charged and I also need to breath; I really need to go out, to walk quickly, I need to get some air. (ID4, daughter, 63 years)

#### 3.2.3 Theme 3: New ways of caring for people with dementia during COVID‐19

##### Adaptation of community‐based services in a digital format

Community‐based services included in this study introduced remote care activities in May 2020. Carers’ devices were mainly mobiles but included tablets and personal computers also. The most used application was Whatsapp followed by Skype, Google Duo, and Meet.

In addition to phone calls, three alternative modalities were adopted. The first one consisted of pen‐and‐paper activities which were sent by staff to carers via email, with a frequency range of between 15 days to a month.

During the COVID‐19, the center sent handouts of about 15 pages with exercises that I printed for him. Among them, there were exercises like crossword puzzles, or even drawings to color aimed at stimulating creative imagination. The center sent them to all attendants at the meeting center. (ID21, daughter, 53 years)

The second modality was represented by pre‐recorded videos sent by staff to carers once a week. Activities were similar to those carried out face‐to‐face, such as low‐impact exercise and music therapy. Some videos even included a personalised message by a staff member for the person living with dementia with his/her name mentioned at the beginning of the video.

They sent us—and still do it—weekly videos by therapists with various exercises, and then they told us to let the person make them according to its abilities. For example, I tried to let my mum do something, but she could only join and follow music therapy. The therapist was playing the guitar and was sending us songs for my mum to follow. Then, she was also talking to us as if she was present. (ID13, daughter, 52 years)

The third modality consisted of videoconferencing to deliver patient support groups, physical and cognitive stimulation activities. They were carried out by a psychologist weekly, and involved small groups (maximum three people living with dementia) or the dyad (care recipient‐informal carer) only. Dedicated chat and support groups for carers were delivered via videoconferencing as well.

The psychologist sent us a message for the appointment, including day and time, and a multiscreen call came. There were two patients including my mum and the psychologist, so that the patient could also see another "colleague" beyond the psychologist. They discussed about the situation and they shared their emotions and feelings. It was a little help, but quite positive. (ID6, son, 52 years)

##### Perceived effects of remote care and support

Phone calls and videoconferencing allowed the continuity of relationships with services staff and users. They were fundamental for people living with dementia, due to their need for relationship continuity and their difficulty maintaining these relationships due to their memory loss. Moreover, remote activities provided participants with practical and emotional support to manage the everyday difficulties that were exacerbated by the pandemic, thus reducing their feelings of isolation and loneliness.

I appreciated the continuity these things were done with and shared with everyone. Thanks to video calls this contact has never been broken, it was not a physical contact but through technology it has never been broken, so this continuity was fundamental, like all things if you do not cultivate them they do not grow. We tried to keep what we had built previously, efforts have been made by psychologists, by ourselves as family members, and also by our relatives who made few friends over years. Continuity is fundamental, as time flows the person will forget more and more, this is a tragedy, it needs a continuous stimulus that allows you to be on the clock. (ID10, daughter, 56 years)

Remote activities broke a daily routine characterised by a lack of stimulation while providing, at least to some extent, people living with dementia with specialist support and care. Many participants noticed that their relatives’ mood improved during videoconferencing where they appeared less nervous and happy.

I can see she is calm and smiling. Indeed, being always at home with the same people, makes it more difficult for her to smile, especially because she cannot go outside. As for video calls, there was a different person who broke the routine by coming home even by phone. This took her mind off things. Even though just a couple of hours a week does not change much, it's something different. I saw her showing a more peaceful face when she was on call. (ID2, daughter, 60 years)

##### Unmet care needs

Many participants complained about the low frequency of remote activities. Due to strict restrictions on social interaction, multiple videoconferencing sessions per week could better meet their relative's need for social contact and support.

[…] If you cannot go to visit your neighbors and see many people, a frequency of 3 to 4 times a week is necessary. As long as it is face‐to‐face or remote, face‐to‐face would even be better, but if this is not possible because of lockdown, at least a video call would be nice. Taking all safety measures to protect the person with dementia, to whom you cannot even explain why, it is very important that he still sees and talks to someone. (ID14, daughter, 61 years)

Participants also mentioned the need for a combination of virtual and practical support at home, which was perceived vital in the difficult times of COVID‐19. Specifically, home visits by health and social care professionals could provide human contact and cognitively and socially stimulate the person living with dementia better, while allowing respite care hours for the informal carer.

I also had to hire a paid carer because it is different to have them away from home 4, 5 times a week, from being at home with them all the time. In short, an external support is needed because otherwise the workload is too heavy. (ID17, daughter, 62 years)

Remote activities indeed required carers’ supervision and assistance due to people living with dementia's digital illiteracy. The latter, which is quite common among older people, was even more marked on people living with dementia, due to their condition.

For those who were not born in the digital age including myself, it can be a foreign and detached world. I had to stay close to her, because otherwise she did not understand anything. (ID1, daughter, 54 years)

## 4 DISCUSSION

This study explored the experience of COVID‐19 restrictions on the lives of community‐dwelling people living with dementia and their informal carers in Italy. Specifically, we focused on access to community‐based services and adopted solutions to provide support and care following the pandemic outbreak. Overall, our findings confirm and extend previous evidence on the detrimental effects of social isolation and the sudden lack of care on informal carers and people living with dementia's cognitive and physical health, and emotional well‐being (Azarpazhooh et al., 2020; Brown et al., 2020; Giebel, Cannon, et al., 2021).

COVID‐19 restrictive measures and the very limited access to health and social support services led to multiple disruptions on people living with dementia's daily lives, thus exacerbating pre‐existing vulnerabilities. Social care and social support services offer a range of psychosocial interventions that help to maintain or improve people with dementia's physical and cognitive functions, interpersonal relationships and quality of life, thus supporting them to live well with their condition while delaying their institutionalisation (Chirico, Chattat, et al., 2021; McDermott et al., 2019; Ottoboni et al., 2021). With the onset of the pandemic, face to‐face social care and social support services were suddenly interrupted and restrictions on social distancing were introduced, thus leading to people with dementia's impaired health, increased/worsened behavioural and psychological symptoms and, therefore, greater dependence on carers.

Informal carers, who already provide the majority of care during normal times, had to spend more hours with their relatives and felt trapped in their own home, with no chance of relief from the caring role and uncertainty about the duration of restrictions. Moreover, the intensity of care increased, as people with dementia were less autonomous and more difficult to manage than before the pandemic, with increasing apathy and lack of interest, motivation and emotions. With informal carers already experiencing high levels of distress before COVID‐19 (Sutcliffe et al., 2017), there is increasing evidence that the forced isolation and service closures magnified carer burden, thus reducing their confidence in their own efficacy as carers (Zucca et al., 2021). Consequently, as carers could be affected by the pandemic even more than patients themselves, it is important to provide them with timely and ongoing instrumental and emotional support to adapt to COVID‐19‐related challenges (Greenberg et al., 2020; Tsapanou et al., 2021). Better support for carers would likely benefit patient care, while avoiding the worsening of care recipient‐care provider relationships and the potential long‐term effects of the pandemic upon already vulnerable populations (Altieri & Santangelo, 2021; Carpinelli Mazzi et al., 2020; Giebel, Cannon, et al., 2021). Comprehensive care policies are therefore fundamental for minimising the adverse outcomes of social isolation and service closures on people with dementia and carers, thus enabling them to adapt as best as possible to pandemic changes.

To our knowledge, in the context of COVID‐19, no studies have explored if and what alternative forms of support and care have been implemented by community‐based services in Italy. While some evidence reported increased mortality rates of older nursing home residents in Italy during the pandemic compared to non‐pandemic times (de Girolamo et al., 2020), no evidence has focused on access to dementia care in the community in Italy prior to this study. Interestingly, the services included in our study were able to organise and provide remote activities nearly 2 months after the pandemic outbreak. These activities were designed to mirror those carried out before the pandemic. They principally consisted of pen‐and‐paper exercises sent by email, pre‐recorded videos or live sessions of psychosocial interventions via videoconferencing.

Remote activities were particularly important as they guaranteed the continuity of relationships with known people (i.e., staff and service users) and of care. Based on our findings, they overall provided participants with emotional and social benefits allowing, in particular, the maintenance of those social relationships that people with dementia built with effort over time. It is well known that, due to their condition, people with dementia need stimulation and a daily routine accompanied by a stable social environment (Keng et al., 2020). This was even more important during the pandemic when people were forced to stay in their own homes and could not even walk outside. Hence, remote support and care helped to mitigate, at least to some extent, the increasing feelings of isolation and loneliness that negatively impact on the mental health of older adults, and of people with dementia particularly (Muntsant & Giménez‐Llort, 2020).

With reference to remote activities, it is also important to highlight that our sample consisted of adult children and did not include spouses or friends. This should be taken into account when interpreting our findings since children often tend to be more digitally competent than their elderly parents (Arighi et al., 2021), and thus often able to support them during remote activities better, as found in our study. Therefore, remote activities should be carefully designed taking into account older adults’ low digital literacy and people with dementia's potential difficulties to use technology (Ramsetty & Adams, 2020; Seifert et al., 2021; Wu et al., 2015). Training packages to improve older adults’ skills and their attitude towards technology, along with appropriate digital environments, could decrease the risk of social inequity and injustice for already vulnerable populations (Manca et al., 2020; O’Shea, 2020).

Nevertheless, based on our findings, the adaptation of services in a digital format did not fully meet people with dementia and carers’ needs of emotional and practical support, which had further increased during the pandemic. More digital support and a combination of virtual and face‐to‐face activities could better counterbalance the multiple adverse indirect outcomes of COVID‐19. Specifically, home visits by professionals would cognitively and socially stimulate people with dementia, while providing temporary relief for informal carers. This is also particularly important since there is evidence that human touch is vital for people with dementia, thus increasing their well‐being as well as reducing their behavioural and psychological symptoms (Wu et al., 2017). Despite these limitations, benefits associated with remote activities, such as for people with reduced mobility or living in remote and rural areas, maybe maximised and integrated into everyday care, as to provide new ways of supporting people with dementia to live well with their condition during both normal and exceptional times.

### 4.1 Limitations and future directions

This study explored the experiences of COVID‐19 public health restrictions on the lives of community‐dwelling people with dementia and their informal carers in Italy. Our findings may be expanded on in future studies including people with dementia as well as their spouses and/or friends. Future research could also include data from other regions than Emilia Romagna, which is a relatively advanced region in terms of the health and social care system in Italy with its own regional fund for non‐self‐sufficient people. It would be also interesting to investigate, a year and a half after the pandemic outbreak, the long‐term effects of COVID‐19 on people with dementia, as well as their abilities to cope with a remote social world. Since services have likely adapted better now, this would also provide insight into the potential of remote activities to be integrated into everyday care even in non‐pandemic circumstances.

## 5 CONCLUSIONS

Findings of this study highlight the large burden of the COVID‐19 pandemic on people living with dementia and their informal carers. Care policies and public health measures should be designed carefully balancing safety needs and the physical, psychological and social needs of people living with dementia. Their needs increased during the pandemic, thus requiring more specialist support and care for an already vulnerable population. Within a holistic care approach, social care services need to be enabled better to guarantee high‐quality care across different regions, thus minimising the detrimental effects of prolonged social isolation and temporary closure of in‐person services on people with dementia and carers. The results of this study not only have implications for social care in Italy but also on a global context too.

## CONFLICT OF INTEREST

The authors report no conflict of interest.

## AUTHOR CONTRIBUTIONS

CG designed the study with MG in the UK and led the project. IC and GO collected data in Italy, analysed data and wrote drafts of the manuscript. Italian team members IC, GO, AP, MV, VDE, RC contributed to data analysis, and all the authors read through drafts of the manuscript and approved the final manuscript.

## Data Availability

No additional data available.

## ENDNOTE

^1^
Curfew, masks, social distancing, hand washing.
